# Cytotoxic Activity and Metabolic Profiling of Fifteen *Euphorbia* Species

**DOI:** 10.3390/metabo11010015

**Published:** 2020-12-29

**Authors:** Seham S. El-Hawary, Rabab Mohammed, Ahmed F. Tawfike, Nadia M. Lithy, Sameh Fekry AbouZid, Mohamed N. Amin, Usama Ramadan Abdelmohsen, Elham Amin

**Affiliations:** 1 Department of Pharmacognosy, Faculty of Pharmacy, Cairo University, Giza 12613, Egypt; seham.elhawary@yahoo.com; 2 Department of Pharmacognosy, Faculty of Pharmacy, Beni-Suef University, Beni-Suef 62514, Egypt; rmwork06@yahoo.com (R.M.); sameh.zaid@pharm.bsu.edu.eg (S.F.A.); 3 Department of Pharmacognosy, Faculty of Pharmacy, Helwan University, Cairo 11795, Egypt; ahmed.tawfike@rothamsted.ac.uk; 4 Molecular Discovery Group, Department of Computational and Analytical Science, Rothamsted Research, Harpenden AL5 2JQ, UK; 5 Department of Pharmacognosy, Faculty of Pharmacy, Nahda University, Beni-Suef 62521, Egypt; nadia.lithy@nub.edu.eg; 6 Department of Pharmacognosy, Faculty of Pharmacy, Heliopolis University, Cairo 11785, Egypt; 7 Department of Biochemistry, Faculty of Pharmacy, Mansoura University, Mansoura 35516, Egypt; dr_mohamednasr@mans.edu.eg; 8 Department of Pharmacognosy, Faculty of Pharmacy, Minia University, Minia 61519, Egypt; 9 Department of Pharmacognosy, Faculty of Pharmacy, Deraya University, Universities Zone, New Minia City 61111, Egypt; 10 Department of Medicinal Chemistry and Pharmacognosy, College of Pharmacy, Qassim University, Buraidah 52571, Saudi Arabia

**Keywords:** *Euphorbia*, cytotoxic activity, metabolic profiling, LC-HRMS, PCA

## Abstract

*Euphorbia* is a large genus of flowering plants with a great diversity in metabolic pattern. Testing the cytotoxic potential of fifteen *Euphorbia* species revealed highest activity of *E. officinarum* L. against human colon adenocarcinoma (CACO2) cell line (IC_50_ 7.2 µM) and of *E. lactea* Haw. against human hepatoma (HepG2) and human breast adenocarcinoma (MCF-7) cell lines (IC_50_ 5.2 and 5.1 µM, respectively). Additionally, metabolic profiling of the fifteen tested species, using LC-HRMS, for dereplication purposes, led to the annotation of 44 natural compounds. Among the annotated compounds, diterpenoids represent the major class. Dereplication approach and multivariate data analysis are adopted in order to annotate the compounds responsible for the detected cytotoxic activity. Results of Principle component analysis (PCA) come in a great accordance with results of biological testing, which emphasized the cytotoxic properties of *E. lactea* Haw. A similarity correlation network showed that the two compounds with the molecular formula C_16_H_18_O_8_ and C_20_H_30_O_10_, are responsible for cytotoxic activity against MCF-7 and HepG2 cell lines. Similarly, the compound with molecular formula C_18_H_35_NO correlates with cytotoxic activity against CACO2.

## 1. Introduction

Cancer represents one of the most lethal diseases worldwide. Cancer treatments have adverse effects. Moreover, not all tumors react in the same way to the treatment. Natural products are considered as a promising approach to cancer control and management [1]. Several studies investigated the cytotoxic potential of phytoconstituents against variable cancer cell lines [2].

Genus *Euphorbia* belongs to family Euphorbiaceae, spurge family, which is composed of about 50 tribes, 300 genera, and 8000 species [3]. *Euphorbia* is the third largest genus of flowering plants, only after *Astragalus* (Fabaceae) and *Psychotria* (Rubiaceae) with approximately 2160 species [4]. Members of this genus are characterized by the production of a milky irritant latex which is exuded when they are injured. *Euphorbia* L. are widely distributed throughout both tropical and temperate regions and range in morphology from small, annual or perennial herbaceous plants to woody shrubs, trees and even large desert succulents [3,5].

Different *Euphorbia* species are used traditionally for the treatment of digestive system disorders as diarrhea, jaundice, constipation, colic and indigestion [4]. Furthermore, they are also used for the treatment of skin diseases, gonorrhea, migraines, intestinal parasites, inhibition of HIV-1 viral infection, warts and for mediating pain due to their antipyretic and analgesic activity [5].

*Euphorbia* exhibited a wide variety of compounds with diverse pharmaceutical activities. Diterpenes, triterpenes, steroids, phenolics and flavonoids are among secondary metabolites isolated from genus *Euphorbia* [5]. In the past few years, many studies have been performed on the cytotoxic activity of *Euphorbia* diterpenes as they proved to have moderate or strong anti-proliferative activity due to the lactone structures. *Euphorbia* diterpenes also reported to own potent antineoplastic activity towards various cancer cell lines (e.g., chronic myeloid leukemia and nasopharyngeal, pancreatic, lung, ovarian, and colon carcinomas) [3].

Plants have a great challenge in metabolomics due to the high chemical and physical diversity of their metabolites [6]. Furthermore, Metabolomics is being applied to identify and biotechnologically optimize the production of pharmacologically active secondary metabolites [7]. In this framework, liquid-chromatography coupled to high resolution mass spectrometry (LC-HRMS) is performed and, by untargeted data-dependent MS/MS experiments, much information on the chemical composition of crude extracts can be created [8]. This interesting task cannot be achieved by a single analytical technique rather several analytical platforms are needed [9]. The dereplication approach and multivariate data analysis are used in order to identify compounds in a mixture responsible for the anti-proliferative effects of plant extracts, and provide a better understanding of the mechanisms of action of medicinal plants [10]. Principle component analysis (PCA) is an unsupervised clustering method requiring no knowledge of the dataset and acts to reduce the dimensionality of multivariate data while preserving most of the variance within [11]. 

Consequently, this study is designed to investigate the cytotoxic potential of fifteen *Euphorbia* species against three cancer cell lines, i.e., HepG2 (human hepatoma), MCF-7 (Human breast adenocarcinoma), and CACO2 (human colon adenocarcinoma) cells. Moreover, metabolic profiling tools and dereplication processes are adopted to investigate the differences in secondary metabolite pattern between the 15 species and annotate the compounds responsible for the tested anti-proliferative activity of the tested *Euphorbia* species 

## 2. Results

### 2.1. Cytotoxic Activity

The cytotoxic activity of fifteen *Euphorbia* species against three cancer cell lines was evaluated (Table 1). Results reveal that five *Euphorbia* species display activity against HepG2 where *E. lactea* Haw. and *E. obesa* Hook. are the most active (IC_50_ 5.2 and 6.3 µg/mL, respectively). Moreover, five species are active against MCF-7 where *E. lactea* Haw. and *E. grandialata* R.A. Dyer exhibit highest activity (IC_50_ 5.1 and 7.5 µg/mL, respectively). On the other hand, eight *Euphorbia* species show cytotoxic activity against CACO2 where *E. officinarum* L. and *E. royleana* Boiss. are the most active (IC_50_ 7.2 and 9.1 µg/mL, respectively). Among fifteen *Euphorbia*, three species, *E. tirucalli* L., *E. horrida* Boiss., and *E. ingens* E. Mey. are inactive against the tested cell lines.

### 2.2. LC-HR/MS Analysis

**Figure 1:**
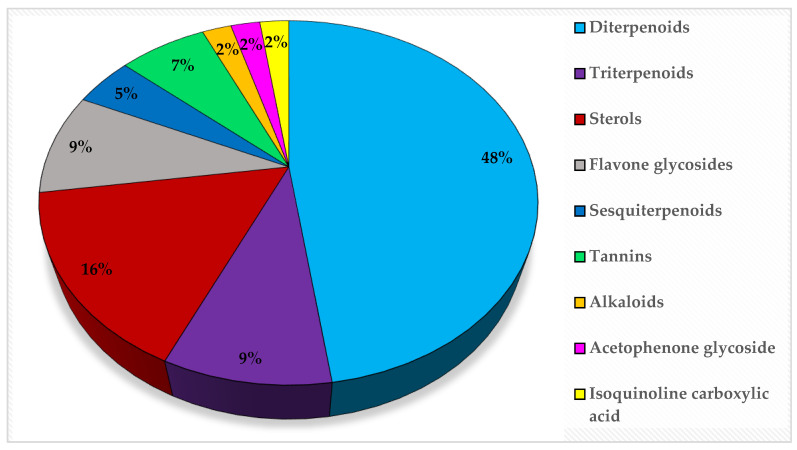
Percentage of different classes of metabolites distributed in the tested 15 *Euphorbia* species.

Metabolic profiling of 15 *Euphorbia* species by LC-HR-MS for dereplication purposes has resulted in the characterization of a variety of metabolites, of which diterpenes are predominant. The dereplication study of the metabolites (Table 2) using the Dictionary of Natural Products (DNP) database followed by chemotaxonomic filtration resulted to the characterization of 44 natural compounds from the 15 studied *Euphorbia* species. The annotated compounds can be classified into: diterpenoids (21 compounds), sterols (seven compounds), triterpenoids (four compounds), flavone glycosides (four compounds), tannins (three compounds), sesquiterpenoids (two compounds), alkaloid (one compound), acetophenone glycosides (one compound), and isoquinoline-carboxylic acid (one compound). As illustrated in (Figure 1), diterpenoids represent the most predominant chemical class in the tested species.

### 2.3. Metabolic and Molecular Correlations Analysis

**Figure 2:**
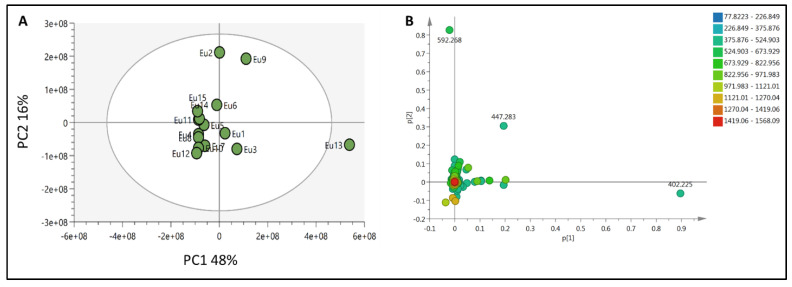
(**A**): PCA score plot for all crude extracts of the tested *Euphorbia* species, (**B**)**:** PCA loading plot showing the distinctive metabolites.

**Figure 3:**
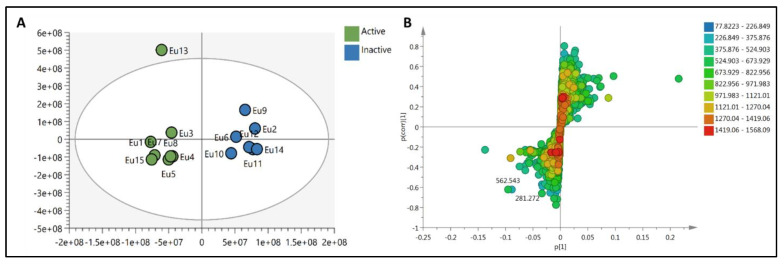
(**A**)**:** The whole molecular network showing the clusters of different features. (**B**)**:** extracted network showing the metabolites highly correlated to the cytotoxic activity against MCF-7 and HPEG2. Nodes are colored in pie chart and molecules grouped according to their concentration in the tested species. Edges are correlation values and the edge’s width is proportional with this value.

**Figure 4:**
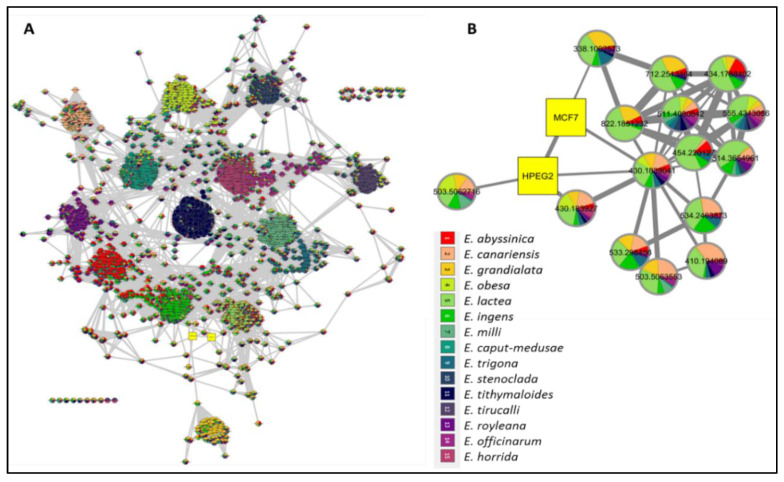
(**A**): OPLS-DA score plot showing a clear separation between (●) CACO2 cytotoxic extracts and (●) inactive ones, (**B**): S-loading plot showing the metabolites highly correlated with CACO2 cytotoxicity.

To provide a holistic coverage of *Euphorbia’s* metabolic profile, the crude extracts of the tested species were analyzed in both positive and negative ion electrospray ionization (ESI) MS modes as changes in ESI polarity can often hinder competitive ionization and suppression effects, revealing otherwise suppressed metabolite signals. This resulted in approximately 3000 molecular ions of both polarities. Principle component analysis (PCA) was applied to the dataset to explore the relative variability and/or similarity of the chemical profiles among the tested species. PCA score plot (Figure 2A) exhibited a respective total variance of 48% and 16% for PC1 and PC2, and highlighted the outlying of EU2, EU9, and EU13 corresponding to the crude extracts of *E*. *caput*-*medusae* L., *E. horrida* Boiss. and *E*. *lactea* Haw., respectively. The dispersal of the former extracts revealed for their unique chemical profiles which led to probe the metabolites contributed to such segregation. PCA loading plot (Figure 2B) demonstrated the discriminatory molecules at m/z (retention time in minutes) 592.268 [M^+^] (t_R_ 29.13) characteristic for *E*. *caput*-*medusae* L. and *E*. *horrida* Boiss., while *E*. *lactea* Haw. was characterized by 402.225 [M^+^] (t_R_ 24.58). The molecular correlation network uses Pearson correlation coefficient to detect molecules highly correlated with bioactivities (represented by percentage of inhibition against cancer cells). Features (molecules) are connected by edges (correlation values) where an edge’s width is corresponding to the strength of this correlation [12]. The threshold of Pearson correlation coefficient was set to 0.8 and a network of metabolites linked to the bioactivity is depicted in (Figure 3A). The network is mapped so nodes are colored in pie chart according to their concentration in the tested species and labeled with feature’s molecular weight. An extracted network of the molecules directly linked to the bioactivity and their neighbor ions (Figure 3B), showed metabolites at m/z [M^+^] (retention time in minutes) 338.100 (9.40) and 430.184 (10.94) are highly correlated with MCF-7 activity. In addition, the other molecule directly linked to HPEG2 activity at m/z 503.506 [M^+^] (t_R_ = 29.66), was not reported before, that refers to a new chemical structure still to be discovered. The network did not detect any molecules correlated with CACO2 cytotoxic activity, even though the threshold was decreased. To highlight the molecules could be contributed to the cytotoxic activity against CACO2 cancer cell line, an OPLS-DA (orthogonal partial least square discrimination analysis) module was created. OPLS-DA was validated by permutation test. The test showed the original R2 and Q2 values were more than the permuted values and the cumulative value of Q2 is −0.08 (less than zero) which is indicative for the good prediction ability of the model. Discriminatory elements were confirmed by descriptive statistics, i.e., *p*-value<0.05, coefficient of variation (95% confidence limits do not cross zero), and variable importance (VIP values >1). The score plot (Figure 4A) demonstrated a clear separation between the active and inactive extracts with a strong goodness of fit R^2^ = 0.96 and a goodness of prediction Q^2^ = 0.46. The S-loading plot (Figure 4B) is a very useful tool to compare the variable magnitude against its reliability, where axes plotted from the predictive components are the covariance p against the correlation p(cor). The molecules highly correlated with the CACO2 activity were checked and only those with high coefficient of variation, whose 95% confidence level limits not crossing zero, were chosen. The significant metabolite highly correlated with the CACO2 cytotoxicity was at m/z [M]^+^ 281.272 (t_R_ = 29.16). 

## 3. Discussion

### 3.1. Cytotoxic Activity

However, the anticancer activity of *E. lactea* Haw. remains largely unexplored, its methanolic extract exhibits high activity against both HepG2 and MCF-7 cell lines (IC_50_ 5.2 and 5.1 µM, respectively). Previous studies tested the ethanolic extract of *E. lactea* Haw. against another hepatic cancer cell line HEp-2 and the IC_50_ was found to be 89 µg/mL [13]. In addition, it was reported that the hydro-alcoholic extract of *E. lactea* Haw. exhibited cytotoxic and anti-migratory activities toward HN22 cells [14]. Additionally, *E. officinarum* L. and *E. royleana* Boiss. are the most active against CACO2. Interestingly, the cytotoxicity of *E. officinarum* L. hasn’t tested before on any cancer cell lines, and the cytotoxic activity of the hexane extract of *E. royleana* Boiss. was only studied on a potato disc and found to be 61.66% at 800 µg/mL [15]. Current results also show mild cytotoxic activity of *E. trigona* Mill. extract against both MCF-7 and CACO2. The latex of *E. trigona* Mill. was previously tested on HT-29 (colon cancer cell line) and found to be inactive [16]. Notably, among the fifteen tested extracts, three species, namely *E. tirucalli* L., *E. horrida* Boiss., and *E. ingens* E. Mey., are inactive against the three tested cell lines. However, previous studies reported high cytotoxic activity of the butanol extract of *E. tirucalli* L. against MCF-7 [17] as well as moderate activity of high concentration of aqueous extract of the same species (100–150 µg/mL) against human leukocytes [18]. 

### 3.2. LC-HR/MS Analysis

The distribution of the compounds in the 15 *Euphorbia* species reveals the presence of different chemical classes such as terpenoids, sterols, flavonoids and tannins and all of these classes were reported previously in *Euphorbia* species [5,19]. Also, Milliamine J alkaloid (**44**) is detected only in *E. milli* Des Moul. and this was reported before [20]. Additionally, l-methyl-6-hydroxy-l,2,3,4-tetrahydroisoquinoline-3-carboxylic acid (**1**) and the acetophenone ebractelatinoside C (**19**) record the highest concentrations in the same species. 

Basically, diterpenoids, such as jatrophanes, lathyranes, tiglianes, ingenanes, myrsinols nucleus, and those with oxygen-containing functionalities, are the majority among secondary metabolites of the genus *Euphorbia*. About 400 diterpenoids have been isolated from different *Euphorbia* species [21]. This comes in great accordance with our results that detect diterpenes as the main class of constituents in all tested *Euphorbia* species, where four different nuclei, i.e., lathyrane (10 compounds), tigliane (5 compounds), inginane (4 compounds), and jatrophane (2 compounds), are detected. Furthermore, inginane nucleus represents the highest concentration compared to other nuclei where compound (**21**) ingenol-3-angelate-5,20-diacetate is detected in high concentration especially in *E. abyssinica* J.F. Gmel., followed by compound (**25**), 17-hydroxyingenol-17-benzoate-20-angelate, that is highly represented in *E. horrida* Boiss. Similarly, a study of the diterpenoid ester content of *E. cupanii*, using liquid chromatography coupled to tandem mass spectrometry and molecular networking coupled to unsupervised substructure annotation (MS2LDA) was recently published and showed the presence of premyrsinane/myrsinane diterpene esters [22].

Herein, the sesquiterpene supinaionoside A (**3**), is the leading among other annonated sesquiterpenes and is detected in *E. abyssinica* J.F. Gmel. for the first time. On the other hand, triterpenes have been frequently reported in *Euphorbia* species [5]. Among the detected triterpenoids, euphorbiane (**6**) and canaric acid (**9**) exist in high concentration in *E. milli* Des Moul. 

Considering sterol content, 24-hydroperoxytirucalla-8,25-dien-3β-ol-7-one (**18**) is recorded in the highest concentration in *E. lactea* Haw. and *E. ingens* E. Mey. Also, many sterols were identified in *E. ingens* [23]. Regarding flavonoids, rhamnetin-3-α-arabinofuranoside (**12**) is the main flavonoid detected and recorded in high concentration in almost all the fifteen tested species, a result that agrees with previous report about the prevalence of rhamnetin glycosides within the Euphorbiaceae [24]. In the same context, recent LC-DAD-MS^n^ fingerprint of the phenolics of *E. hirta, E. heterophylla* and *E. convolvuloides* concluded the presence of flavonoids, coumarins and phenolic acids [25]. Tannins, specifically; helioscopinin B (**38**) and 3,3’,4-tri-*O*-methyl-4’-*O*-rutinosyl-ellagic acid (**42**) are detected in high concentration in *E. stenoclada* Baill. and *E. abyssinica* J.F. Gmel., respectively. 

### 3.3. Metabolic and Molecular Correlations Analysis

The dereplication study revealed that molecule at m/z [M+] 592.268 is corresponding to molecular formula (C_35_H_36_N_4_O_5_) which may be phaeophorbide A that is a chlorophyll degradation product formed by enzymic hydrolysis of phaeophytin A by chlorophyllase. More interestingly, signal at m/z 402.225 is equivalent to molecular formula C_20_H_34_O_8_ that may correspond to either taxane or grayanane type of diterpenoid molecule i.e., pierisformosoid which was reported in the literature for its cytotoxic, analgesic and antifeedant properties [71,72]. The PCA and dereplication results matched with the investigated biological activities of the *Euphorbia’s* crude extracts which emphasized the cytotoxic properties of *E*. *lactea* Haw. The biological investigations revealed the cytotoxic activities of *E*. *lactea* Haw. against HPEG2 and MCF-7 cancer cell lines and *E. officinarum* L. against CACO2 cell line. To pinpoint the molecules mediated for such bioactivities, a similarity correlation network was implemented. The molecules 338.100 (9.40) and 430.184 (10.94) that correlated with MCF-7 were equivalent to molecular formula C_16_H_18_O_8_ and C_20_H_30_O_10_ respectively. In addition, the molecule at 430.184 was further correlated to the cytotoxic activity against HPEG2. Moreover, the correlated metabolite with the CACO2 cytotoxicity 281.272 (t_R_ = 29.16) that was equivalent to C_18_H_35_NO. More amazingly, all these molecular formulae not reported to be isolated previously from genus *Euphorbia* and need further investigation. In summary, metabolomics was a powerful tool that gave a shorter access to the bioactive metabolites could mediate for the demonstrated cytotoxicity of *Euphorbia*’s extracts against MCF-7, HPEG2 and CACO2 cancer cell line. Moreover, the dereplication study relied on a precise molecular formula prediction along with ^1^HNMR to minimize the number of hits per each molecular formula, leading to the tentative identification of the top hits [73]. 

## 4. Materials and Methods 

### 4.1. Plant Material

The aerial non-flowering parts of fifteen *Euphorbia* species were collected during October 2018 from Helal Cactus farm, Abdel Samad village, El-Mansuriya, Giza, Egypt. All the collected species consisted mainly of stems and leaves. The species were identified and authenticated by Prof. Dr. Abdel-Halim Mohammed; Professor of Agriculture, Flora department, Agricultural museum, Dokki, Giza, Egypt. Fresh plants were cut into small pieces and directly macerated in methanol 80%.

### 4.2. Methanolic Extracts for Metabolic and Cytotoxic Studies

Methanolic extracts were prepared by maceration of 50 g fresh samples in (3 × 200 mL) 80% methanol (Sigma-Aldrich, Darmstadt, Germany). The extracts were separately filtered and evaporated to dryness using rotatory evaporator. Dried extracts were stored at 4 °C.

### 4.3. Cytotoxic Activity

Cytotoxicity of the methanolic extracts was evaluated in cell lines using the 3-(4,5-dimethylthiazol-2-yl)-2,5-diphenyltetrazolium bromide (MTT) assay [74,75]. HepG2 (human hepatoma), MCF-7 (Human breast adenocarcinoma), and CACO2 (human colon adenocarcinoma) cells were maintained in RPMI medium (Merck, Darmstadt, Germany), supplemented with 10% fetal bovine serum (FBS). Cancer cells were cultured at 37 °C, 5% (*v/v*) CO_2_ in RPMI1640 medium, supplemented with 5% (*v/v*) fetal bovine serum (FBS), 1% (*w/v*) L-glutamine, 1% sodium pyruvate and 0.4% (*w/v*) antibiotics (50 U/mL penicillin, 50 mg/mL streptomycin). Cells were obtained from the American Type Culture Collection (ATCC, Rockville, MD, USA; HPACC, Salisbury, UK) and routinely sub-cultured twice per week. Alcoholic extracts were dissolved in DMSO at a concentration of 0.05 g/0.5 mL as a stock solution and filtered to remove any particulate matter. Further dilutions were made in culture medium. DMSO used for the assay was of ACS reagent grade from Sigma Aldrich (Darmstadt, Germany). The water used was reverse osmosis water purified using a Millipore cartridge filter. The MTT (3-(4,5-dimethylthiazol-2-yl)-2,5diphenyltetrazolium bromide) substance, and all the other reagents and substances were obtained commercially (Sigma Aldrich, USA). The glass vials (2 mL) utilized were Fisher-brand with Titeseal closure (Fisher Scientific). To normalize cell viability values, each plate included a triplicate of cells treated with the various methanolic extracts carrier on DMSO to define 100% viable cells as well as a triplicate of cells incubated with a cytotoxic mixture (200 ng/mL Tumor Necrosis Factor TNF, 200 ng/mL CD95L (Fas ligand), 200 ng/mL TRAIL (TNF-related apoptosis-inducing ligand), 25 g/mL CHX (Cycloheximide), 1% (*w/v*) sodium azide) to define maximal cell death and thus 0% viability. The viable cells produced a dark blue formazan product, whereas no such staining was formed in the dead cells. All samples were transferred to a 96-well plate and absorbance was measured at 570 nm using a SpectraMax plus Microplate Reader (Molecular Devices, CA, USA). The cell viability was expressed relative to the untreated control cells. All other viability values were normalized according to the averages of these triplicates and analyzed by the Graph Pad Prism 5 software (La Jolla, CA, USA), 50%. 5-Flurouracil was used as a positive control.

### 4.4. LC-HR/MS Analysis

One mg of each extract (of the fifteen species) was weighted using sensitive electric balance (Sartorius, type 1712, Germany) and dissolved in one mL HPLC grade methanol then it was analyzed on an Acquity Ultra Performance Liquid Chromatography system coupled to a Synapt G2 HDMS quadrupole time-of-flight hybrid mass spectrometer (Waters, Milford, CT, USA). The HPLC column was an ACE (ACE, Mainz, Germany) C18, 75 mm × 3.0 mm, 5 µm column. The mobile phase consisted of HPLC grade water (A) that was obtained in-house from a direct Q-3 water purification system (Millipore, Watford, UK) and acetonitrile (B) with 0.1% formic acid in each solvent. All reagents were of analytical grade and were purchased (Fisher Scientific, Hemel Hempstead, UK). The gradient program started with 10% B linearly increased to 100% B at a flow rate of 300 µL/min for 30 min and remained isocratic for 5 min before linearly decreasing back to 10% B in 1 min. The column was then re-equilibrated with 10% B for 9 min before the next injection. The total analysis time for each sample was 45 min. The injection volume was 10 µL, and the tray temperature was maintained at 12 °C. High resolution mass spectrometry was carried out in both positive and negative ESI ionization modes with a spray voltage at 4.5 kV and capillary temperature at 320 °C. The mass range was set from m/z 150–1500. Both negative and positive ionization switch modes were used to include the highest number of metabolites from the investigated methanol extracts subjected to LC–HR-ESIMS analysis. The dereplication was achieved for each m/z ion peak with metabolites recorded in the customized databases based on established parameters (m/z threshold of ±3 ppm and retention time) [76], which provided a high level of confidence in metabolites identity. Consequently, the number of the remaining unknown metabolites in each species was refine. The raw data were processed, aligned, and merged into one dataset according to the method previously developed in our lab [77,78].

## 5. Conclusions

The present study highlighted the cytotoxic activity and metabolic profiling of fifteen *Euphorbia* species where *E. lactea* Haw. shows the highest cytotoxic activity against HepG2 and MCF-7 (IC_50_ 5.2 and 5.1, respectively). On the other hand, *E. officinarum* L. is the most active against CACO2 IC_50_ 7.2. The molecular interaction network is implemented in order to correlate the chemical and biological profiles. Interestingly, molecule detected at m/z 503.506 [M^+^] (t_R_ = 29.66), directly linked to HepG2 activity, was not reported before, suggesting that a new chemical structure still to be discovered. Also, the molecular correlations analysis reveals for the unique chemical profiles of *E*. *caput*-*medusae* L.*, E*. *horrida* Boiss.*,* and *E*. *lactea* Haw. where means of 592.268 [M^+^] (t_R_ 29.13) is characteristic for *E*. *caput*-*medusae* L. and *E*. *horrida* Boiss. while 402.225 [M^+^] (t_R_ 24.58) is characterized for *E*. *lactea* Haw.

## Figures and Tables

**Table 1:** IC_50_ µM values of methanolic *Euphorbia* extracts in different cancer cell lines.

Sample Code	HEPG2	MCF-7	CACO2
*E. abyssinica* J.F. Gmel.	--	--	11.3
*E. caput-medusae* L.	--	--	17.2
*E. trigona* Mill.	--	16.1	15.6
*E. stenoclada* Baill.	19.3	19.5	18.2
*E. tithymaloides* L.	--	--	13.6
*E. tirucalli* L.	--	--	--
*E. royleana* Boiss.	--	--	9.1
*E. officinarum* L.	--	--	7.2
*E. horrida* Boiss.	--	--	--
*E. canariensis* L.	9.8	12.7	--
*E. grandialata* R.A. Dyer	8.4	7.5	--
*E. obesa* Hook.	6.3	--	--
*E. lactea* Haw.	5.2	5.1	--
*E. ingens* E. Mey.	--	--	--
*E. milli* Des Moul.	--	--	9.8

(--) No significant cytotoxicity at the examined concentrations.

**Table 2:** List of secondary metabolites isolated from fifteen *Euphorbia* species.

N.	Compound	Class	Mode	Formula	m/z	Rt	Structure	Source
1	l-Methyl-6-hydroxy-l,2,3,4-tetrahydro-isoquinoline-3-carboxylic acid	Isoquinoline-3-carboxylic acid	-	C_11_H_13_NO_3_	206.0824	10.37	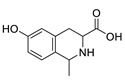	*E. myrsinites* L. [26]
2	4-Deoxyphorbol	Diterpene	+	C_20_H_28_O_5_	349.2006	9.63	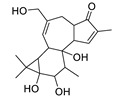	*E. tirucalli* L. [27]
3	Supinaionoside A	Sesquiterpene	-	C_19_H_30_O_9_	401.1817	10.01	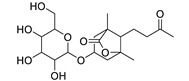	*E. supina* Raf. [28]
4	Euphorbioside B	Sesquiterpene	+	C_19_H_34_O_9_	407.2271	8.12	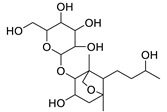	*E. resinifera* Berg. [29]
5	Euphorbosterol	Sterol	+	C_29_H_48_O	413.3775	27.46	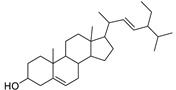	*E. tirucalli* L. [30]
6	Euphorbiane	Triterpene	+	C_30_H_48_O	425.3775	24.48	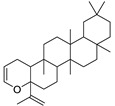	*E. tirucalli* L. [31]
7	Cycloeuphordenol	Sterol	+	C_30_H_50_O	427.3931	26.12	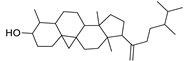	*E. tirucalli* L. [30]
8	Cycloeuphornol	Sterol	+	C_31_H_50_O	439.3931	27.24	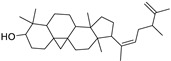	*E. tirucalli* L. [32]
9	Canaric acid(3,4-seco-4(23),20(30)-Lupadien-3-oic acid)	Triterpene	+	C_30_H_48_O_2_	441.3724	27.36	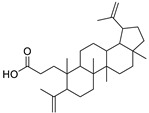	*E. broteri* Daveau. [33]
10	Euphorbinol	Sterol	+	C_31_H_52_O	441.4086	27.33	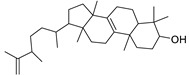	*E. tirucalli* L. [32]
11	3β,7β-Dihydroxy-4α,14α-dimethyl-5α-cholest-8-en-11-one	Sterol	+	C_29_H_48_O_3_	445.3674	27.85	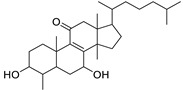	*E. officinarum* L. [34]
12	Rhamnetin-3-α-arabinofuranoside	Flavonoid	+	C_21_H_20_O_11_	449.1074	11.87	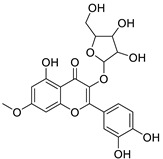	*E. amygdaloides* L. [35]
13	Supinenolone C	Triterpene	+	C_30_H_46_O_3_	455.3517	26.27	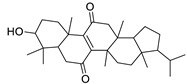	*E. supina* Raf. [36]
14	Euphonerin D	Triterpene	+	C_30_H_48_O_3_	457.3673	20.59	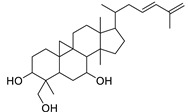	*E. neriifolia* L. [37]
15	3β-Hydroxycycloart-25-ene-24-hydroperoxide	Sterol	+	C_30_H_50_O_3_	459.3829	25.98	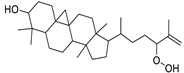	*E. cyparissias* L. [38]
	Cycloart-23-ene-3,25-diol-25-hydroperoxide	Sterol					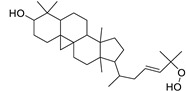	*Tillandsia recurvata* and *Xanthosoma robustum* [39,40]
16	Kaempferol-3-glucuronide	Flavonoid	+	C_21_H_18_O_12_	463.0867	11.92	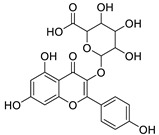	*E. lathyris* L. [41]
	Scutellarin(Breviscapine)	Flavonoid					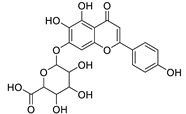	*Scutellaria* spp. and *Erigeron breviscapus* [42,43,44]
17	Ingenol-20-acetate 3-angelate (*Euphorbia* factor Pe1)	Diterpene	+	C_27_H_36_O_7_	473.2531	21.64	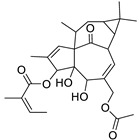	*E. spp* [45]
18	24-Hydroperoxytirucalla-8,25-dien-3β-ol-7-one	Sterol	+	C_30_H_48_O_4_	473.3622	23.34	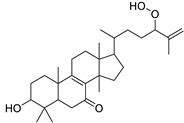	*E. micractina* Boiss. [46]
19	Ebractelatinoside C	Acetophenone	+	C_21_H_30_O_13_	491.1753	10.11	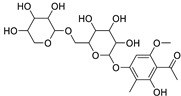	*E. ebracteolata* Hayata [47]
20	Tirucalicine	Diterpene	+	C_27_H_38_O_9_	507.2582	16.79	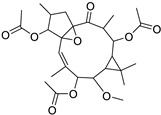	*E. tirucalli* L. [48]
21	Ingenol-3-angelate-5,20-diacetate	Diterpene	+	C_29_H_38_O_8_	515.2636	22.72	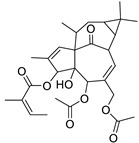	*E. canariensis* L. [49]
22	Euphohelioscopin B	Diterpene	+	C_30_H_42_O_7_	515.2998	24.44	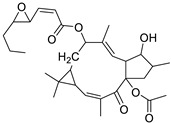	*E. helioscopia* L. [50]
23	16-Angeloyloxy-13α-isobutanoyloxy-4β,9α,7β- trihydroxytiglia-1,5-dien-3-one	Diterpene	+	C_29_H_40_O_8_	517.2792	23.04	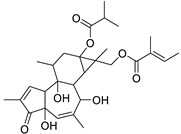	*E. grandicornis* Goebel [51]
	13-*O*-[2-Methyl-2-cis-butenoyl]-16-*O*-isobutyryl -12-desoxy-16-hydroxy-phorbol(*Euphorbia* factor C)	Diterpene					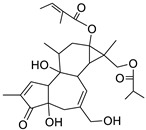	*E. cooperi* N. E. Br. [52]
24	12-*O*-(2Z,4E-Octadienoyl) -phorbol-13-acetate	Diterpene	+	C_30_H_40_O_8_	529.2791	20.37	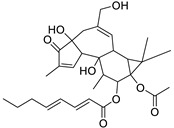	*E. tirucalli* L. [53]
25	17-Hydroxyingenol-17-benzoate-20-angelate	Diterpene	+	C_32_H_38_O_8_	551.2634	22.97	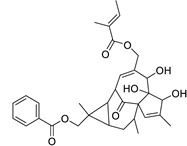	*E. canariensis* L. [49]
26	12-*O*-(2Z,4E,6-Decatrienoyl)-phorbol-13-acetate	Diterpene	+	C_32_H_42_O_8_	555.2949	24.67	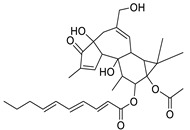	*E. tirucalli* L. [53]
27	12-*O*-Acetylingol-3,8-ditiglate	Diterpene	+	C_32_H_44_O_9_	573.3053	22.48	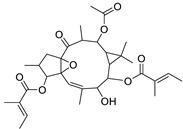	*E. royleana* Boiss. [54]
28	3,7,12-Tri-*O*-acecy1-8-isovaleryl-ingol	Diterpene	+	C_31_H_44_O_10_	577.3000	25.83	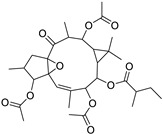	*E. tirucalli* L. [55]
29	5,15,17-Tri-*O*-acetyl-3-*O*-benzoyl-l7-hydroxy-isolathyrol	Diterpene	+	C_33_H_40_O_9_	581.274	22.95	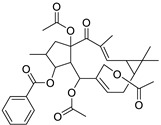	*E. lathyris* L. [56]
30	8-Methoxyingol-7,12-diacetate-3-phenylacetate	Diterpene	+	C_33_H_42_O_9_	583.2896	25.25	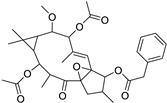	*E. officinarum* L. [57]
31	12-*O*-Acetyl-3-*O*-benzoylingol-8-tiglate	Diterpene	+	C_34_H_42_O_9_	595.2890	23.05	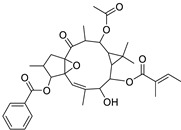	*E. royleana* Boiss. [54]
32	3-*O*-(2,4,6-Decatrienoyl)-16-*O*-angeloyl-ingol(*Euphorbia* factor I5)	Diterpene	+	C_35_H_46_O_8_	595.3260	25.79	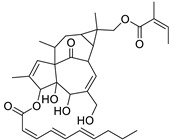	*E. ingens* E. Mey. [58]
33	12-*O*-(2,4,6,8-Tetradecatetraenoyl)-phorbol-13-acetate	Diterpene	+	C_36_H_48_O_8_	609.3417	26.12	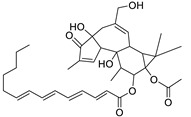	*E. tirucalli* L. [53]
34	Quercetin-3-*O*-β-D-glucopyranosyl-(1-4)-*O*-α-L-rhamnopyranoside	Flavonoid	+	C_27_H_30_O_16_	611.1599	10.83	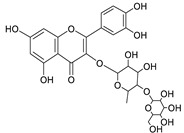	*E. drancunculoides* Lam. [59]
	Vincetoxicoside A	Flavonoid					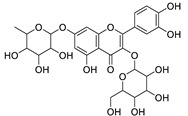	*Tilia* spp.*, Vincetoxicum* spp. and many other plant spp. [60,61,62]
	Rutin	Flavonoid					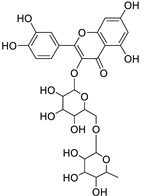	*Ruta graveolens* L. and Many plants [63,64]
35	8,12-*O*-Diacetylingol-3,7-ditiglate	Diterpene	+	C_34_H_46_O_10_	615.3155	24.11	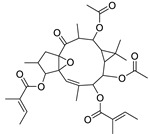	*E. royleana* Boiss. [54]
36	Quercetin-3-*O*-(2’’-galloyl) -β-D-galactopyranoide	Flavonoid	-	C_28_H_24_O_16_	615.0990	10.77	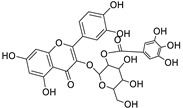	*E. spp.* [65,66]
37	Euphocharacin G	Diterpene	+	C_34_H_45_NO_10_	628.3109	23.20	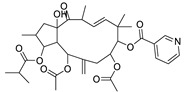	*E. characias* L. [67]
38	Helioscopinin B	Tannin	-	C_27_H_22_O_18_	633.0732	8.99	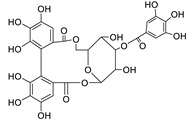	*E. helioscopia* L. [68]
39	2,3-Di-*O*-methylellagic acid-7-*O*-rutinoside	Tannin	+	C_28_H_30_O_17_	639.1548	10.59	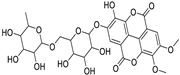	*E. tirucalli* L. [69]
40	Ingol-7,8,12-triacetate-3-(4-methoxyphenyl)-acetate	Diterpene	+	C_35_H_44_O_11_	641.2962	22.71	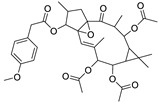	*E. officinarum* L. [57]
41	Euphocharacin K	Diterpene	+	C_35_H_47_NO_10_	642.3267	23.23	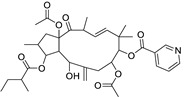	*E. characias* L. [67]
42	3,3’,4-Tri-*O*-methyl-4’-*O*-rutinosyl-ellagic acid	Tannin	+	C_29_H_32_O_17_	653.1704	11.93	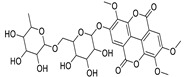	*E. acaulis* Roxb. [70] *E. tirucalli* L. [69]
43	8,12-*O*-Diacetylingol-3,7-dibenzoate	Diterpene	+	C_38_H_42_O_10_	659.2844	23.04	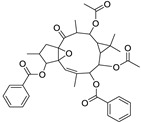	*E. royleana* Boiss. [54]
44	Milliamine J	Alkaloid	+	C_44_H_47_N_3_O_10_	778.3324	26.81	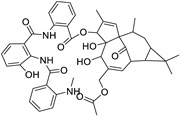	*E. milli* Des Moul. [20]

## Data Availability

All data generated or analyzed during this study are included in this published article.
